# Supplementary material for: Polyketide synthase gene clusters in the ericoid mycorrhizal fungus Oidiodendron maius: genome-wide prediction, expression profiling, and functional characterization of knockout mutants
Source: Front Plant Sci. 2026 Sep 16;17:1924430. doi: 10.3389/fpls.2026.1924430 (PMC13623903; doi:10.3389/fpls.2026.1924430)
Supplement: Supplementary file 1 [file SupplementaryFile1.zip › Supplementary Materials/Supplementary Folder 1.zip!/Oidma1_AssemblyScaffolds_Repeatmasked/knownclusterblast/cluster51/jgi.p_Oidma1_137859_mibig_hits.html]

| MIBiG Protein | Description | MIBiG Cluster | MiBiG Product | % ID | % Coverage | BLAST Score | E-value |
| --- | --- | --- | --- | --- | --- | --- | --- |
| AEN83887.1 | AdaC | BGC0000156 | TAN-1612\_biosynthetic\_gene\_cluster | 59 | 99.2665036675 | 493 | 1.7e-139 |
| ADI24934.1 | VrtH | BGC0000168 | Viridicatumtoxin\_biosynthetic\_gene\_cluster | 58 | 97.5550122249 | 492 | 5e-139 |
| EGD99350.1 | FAD-dependent\_monooxygenase | BGC0001144 | Neosartoricin\_biosynthetic\_gene\_cluster | 55 | 100.244498778 | 460 | 2.1e-129 |
| CBF70383.1 | FAD-dependent\_monooxygenase,\_putative\_(AFU\_orthologue;\_AFUA\_7G00150) | BGC0000684 | Asperthecin\_biosynthetic\_gene\_cluster | 53 | 100.97799511 | 440 | 2.3e-123 |
| AGO65986.1 | putative\_monooxygenase | BGC0000992 | Fusaridione\_A\_biosynthetic\_gene\_cluster | 24 | 90.7090464548 | 63 | 5.8e-10 |
